# Immune checkpoint inhibitors of PD-L1 as cancer therapeutics

**DOI:** 10.1186/s13045-019-0779-5

**Published:** 2019-09-05

**Authors:** Akintunde Akinleye, Zoaib Rasool

**Affiliations:** Department of Internal Medicine, Sovah Health, Danville, VA 24541 USA

**Keywords:** Immune checkpoints, Tumor-infiltrating lymphocytes, Merkel cell carcinoma, T cell dysfunction, Companion diagnostics assays, Non-small cell lung cancer

## Abstract

Since the discovery of immune checkpoint proteins, there has been a special interest in developing antibodies that block programmed cell death 1 receptor (PD-1) and programmed cell death receptor ligand 1 (PD-L1) for a subset of cancer patients. PD-1 signaling negatively regulates T cell-mediated immune responses and serves as a mechanism for tumors to evade an antigen-specific T cell immunologic response. It plays a role in promoting cancer development and progression by enhancing tumor cell survival. With this background, PD-1 signaling represents a valuable therapeutic target for novel and effective cancer immunotherapy. Clinical data shows that blockade of this PD-1 signaling significantly enhance antitumor immunity, produce durable clinical responses, and prolong survival. Currently, there are three FDA-approved PD-L1 inhibitors for various malignancies ranging from non-small cell lung cancer to Merkel cell carcinoma. This review is to summarize many ongoing phase II/III trials of atezolizumab, durvalumab, avelumab, and new PD-L1 inhibitors in clinical developments. In particular, we focus on key trials that paved the pathway to FDA-approved indications for atezolizumab, durvalumab, and avelumab. Despite the popularity and accelerated FDA approval of PD-L1 inhibitors, further considerations into predictive biomarkers, mechanisms of resistance, treatment duration, immune-related toxicities, and PD-L1 expression threshold are needed to optimize anticancer potential in this class of immunotherapy.

## Introduction

Under normal condition, the immune system functions to protect the host against autoimmunity, allergy, and infectious diseases by a series of co-inhibitory and co-stimulatory receptors and their ligands, known as immune checkpoints [1, 2]. Accumulating evidence revealed that tumors use many of these pathways as important mechanisms to escape antitumor immune responses and ultimately progress, disseminate, and metastasize [1, 3]. Among those pathways, programmed cell death 1 (PD-1) and programmed cell death ligand 1 (PD-L1) axis plays a key role in physiological immune homeostasis and putatively served as a means through which cancer cells evade the immune system [4]. The development and application of immune checkpoint inhibitors that block PD-1/PD-L1 interaction result in very durable responses and prolong survival in patients with a wide range of cancers.

## PD-1/PD-L1 axis in health and tumorigenesis

PD-1 also known as CD279 is a 288-amino acid type I transmembrane protein receptor that was discovered as an apoptosis-associated molecule by Tasuku Honjo and colleagues after they cloned the PD-1 gene from immune cell lines undergoing apoptosis in 1992 [5]. It was demonstrated that PD-1 was a negative regulator of immune responses by studying PD-1-deficient mice [6]. The protein is predominantly expressed on antigen-experienced memory T cells in peripheral tissues and less commonly on B cells, activated monocytes, dendritic cells (DCs), and natural killer (NK) cells [1, 5]. It is encoded by the *PDCD1* gene that maps to a 55-kDa DNA fragment that consists of 5 exons located on chromosome 2 [1, 5]. PD-1 is homologous to the CD28 family of protein receptors and composed of immunoglobulin V (IgV)-like extracellular domain that shares sequences identical to other members of the CD28 family proteins, a transmembrane domain, and a cytoplasmic (intracellular) domain of approximately 95 residues that contains 2 phosphorylation sites located in an immunoreceptor tyrosine-based inhibitory motif (ITIM) and an immunoreceptor tyrosine-based switch motif, which, upon phosphorylation, negatively regulates T cell receptor (TCR) signals through phosphorylating Src homology phosphatase-1 (SHP-1) and SHP-22 [1, 5].

**Fig. 1:**
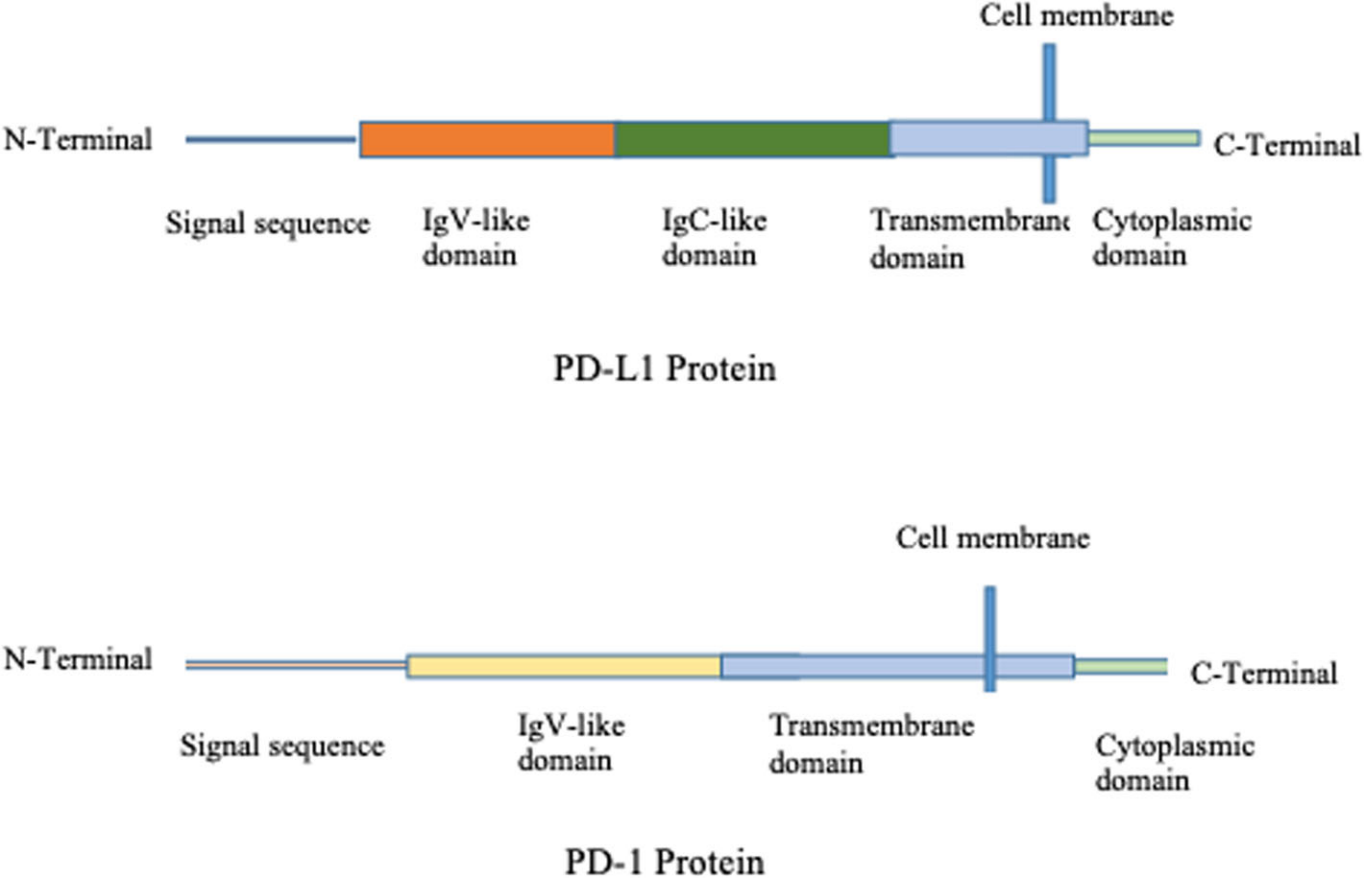
The protein structures of PD-L1 and PD-1. PD-L1 and PD-1 are both transmembrane proteins that interact with each other. PD-L1 mainly contains cytoplasmic domain, transmembrane domain, and two extracellular domains IgV-like and IgC-like. Meanwhile, PD-1 protein only consists of one extracellular domain, transmembrane domain, and cytoplasmic domain

PD-L1 (also known as B7-H1 or CD274) and PD-L2 (also known as B7-DC or CD273) are the two ligands for PD-1 [1, 7]. They are members of the B7 family of type I transmembrane protein receptors [1]. Lieping Chen and colleagues identified and cloned human B7-H1 gene in 1999 and recognized the molecule of having inhibitory effects on T cells by inducing IL-10 [8]. With the discovery of interaction of PD-1 and B7-H1 molecule, it was renamed as PD-L1 [7]. Structurally, PD-L1 is a 290-amino acid protein receptor encoded by *Cd274* gene, comprising of 7 exons, and located on chromosome 9 in humans [1, 5, 7]. It is composed of 2 extracellular domains, IgV- and IgC-like domains; a transmembrane domain; and a cytoplasmic (intracellular) domain as indicated in Fig. 1. The intracellular domain of PD-L1 is short comprising of 30 amino acids, and there is no known function for this domain [1]. The protein is constitutively expressed on many cell types, including antigen-presenting cells (APCs), T cells, B cells, monocytes, and epithelial cells, and is upregulated in a number of cell types after the activation in response to proinflammatory cytokines such as IFNγ and IL4 through signal transducer and activator of transcription-1 (STAT1) and IFN regulatory factor-1 (IRF1) [1, 9].

PD-L2 is encoded by *Pdcd1lg2* gene adjacent to *Cd274* gene separated by 42 kb of intervening genomic DNA in human [1]. It is composed of 273 amino acid residues and comprised of 7 exons which consist of IgV-like domain, IgC-like domain, transmembrane domain, and cytoplasmic (intracellular) domain. In contrast to PD-L1 expression, PD-L2 is restricted largely to APCs and it is inducibly expressed on DCs, macrophages, and bone marrow-derived mast cells [1, 9].

Increasing evidence demonstrates that activation of PD-1/PD-L1 signaling negatively regulates T cell-mediated immune responses in the peripheral tissues to limit effector T cell responses and protect tissues from immune-mediated tissue damage which is also known as peripheral T cell tolerance [1]. PD-1 is not expressed on resting T cells but is inducibly expressed after activation by TCR/antigen-loaded MHC and CD28/B7 interactions [1]. When engaged by its ligands, PD-1 axis dampens T cell responses in several ways largely on cytokine production than on cellular proliferation, with significant effects on IFN-γ, TNF-α, and IL-2 production [1, 9]. PD-1 signaling also exerts its effects on cell differentiation and survival directly by inhibiting early activation events that are positively regulated by CD28 or indirectly through IL-2 [10]. It inhibits kinases involved in T lymphocyte activation via SHP2 phosphatase activity and other signaling pathways [7]. PD-1 ligation inhibits the induction of the cell survival factor Bcl-xL as well as the expression of transcription factors associated with effector cell function, including GATA-3, Tbet, and Eomes [11], and limit autoimmunity at the time of inflammatory response to infections [3, 5, 7, 12]. In addition, PD-1 axis also inhibits lytic activity on activated cells, including B cells and NK cells [13, 14]. More importantly, PD-1 is also highly expressed on regulatory T cells (T_Reg_), where they may be activated and proliferate in the presence of ligands [15] and inhibit, rather than promote, immune responses by expression of the forkhead transcription factor FOXP3, lack of expression of effector cytokines such as IFNγ, and production of inhibitory cytokines such as TGFβ, IL-10, and IL-35.

**Fig. 2:**
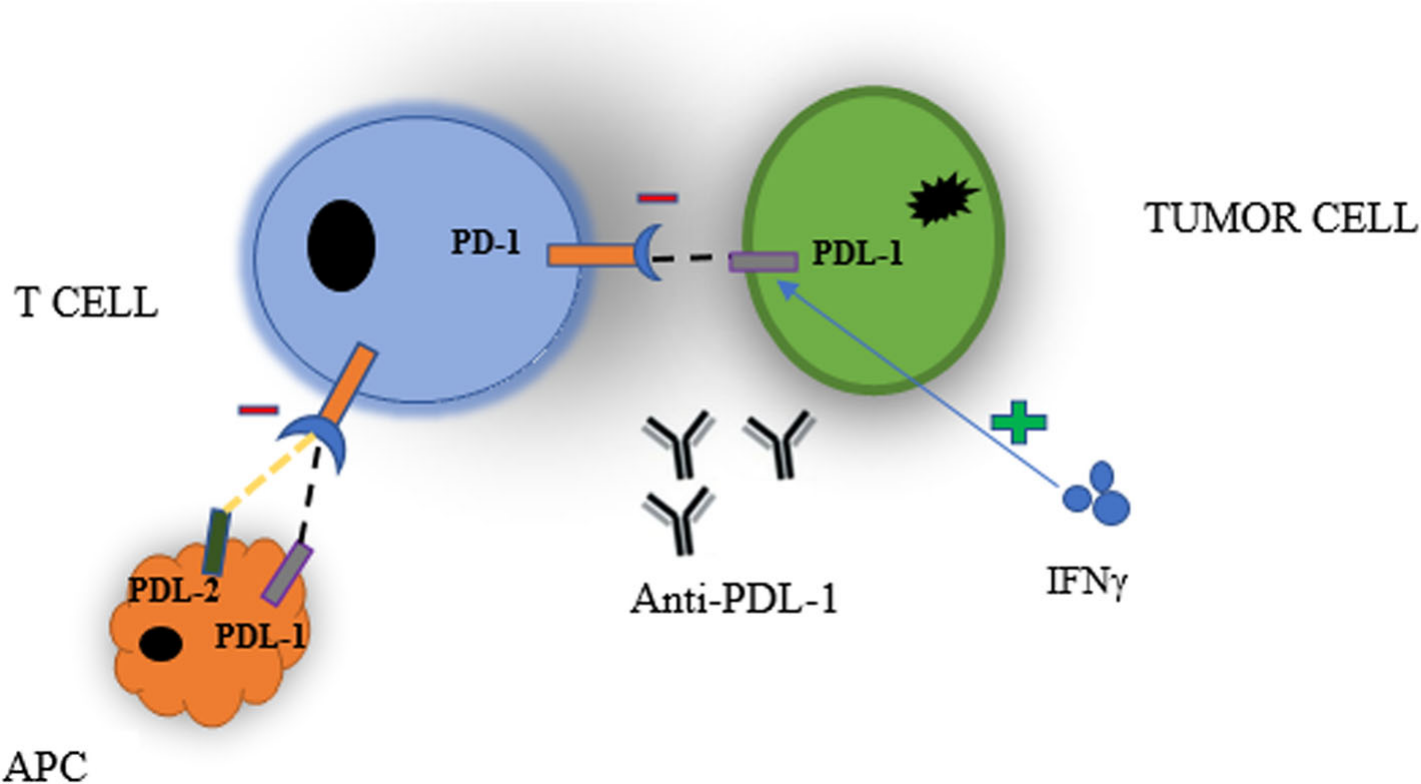
PD-1 and PD-L1 interaction of tumor cells and antigen-presenting cells (APC) with T cells that inhibit immune response. IFNγ help induce or maintain the expression of PD-L1. Anti-PD-L1 inhibits the interaction between PD-1 and PD-L1

Given its pivotal role in preventing autoimmunity and maintenance of peripheral tolerance in normal tissues, it is not surprising that PD-1 signaling pathway can be exploited by tumor cells to evade antitumor immune responses and ultimately progress, disseminate, and metastasize [1, 3, 12]. Recent studies suggest that PD-1 signaling in tumor microenvironment plays a vital role in tumor progression and survival by escaping tumor immune surveillance as shown in Fig. 2. PD-1 is highly expressed in tumor-infiltrating lymphocytes (TILs) in a large proportion among many types of cancers. Of note, PD-1 ligands, especially PD-L1, are constitutively expressed on different types of tumor cells including melanoma and ovarian, lung, and renal carcinomas [16]. Furthermore, PD-L1 expression has been reported to be upregulated in many other human cancers [17] by two general mechanisms, namely innate immune resistance and adaptive immune resistance. By innate immune resistance, PD-L1 expression is upregulated in some tumor cells by constitutive oncogenic signaling through aberrant activation of the PI3K-AKT pathway or chromosomal alterations and amplifications which is found in Hodgkin lymphoma, independent of inflammatory signals in the tumor microenvironment [18, 19]. In contrast, PD-L1 is not constitutively expressed in some tumor cells, but rather is inducibly expressed (i.e., adaptive immune resistance) in response to inflammatory signals elaborated by active antitumor immune responses [20, 21]. Many cytokines can induce or maintain PD-L1 expression; however, IFNγ appears to be the most potent [20–22]. PD-L1 is also commonly expressed on myeloid cells including a subset of macrophages, dendritic cells (DCs), fibroblasts, and endothelial cells in the tumor microenvironment [23–25]. Different types of B cell lymphomas, such as primary mediastinal B cell lymphoma, follicular cell B cell lymphoma, and Hodgkin’s disease, have been reported to express PD-L2 [26].

Overwhelming evidence suggests that interaction of PD-L1/PD-1 in the tumor microenvironment promotes T cell dysfunction, exhaustion, apoptosis, neutralization, and elaboration of IL-10 in a tumor mass creating a state of resistance from cytotoxic T cell (CD8+)-mediated tumor cell killing [17, 27]. It promotes cancer development and progression by enhancing tumor cell proliferation and survival. Recent studies suggest that tumors are highly infiltrated with T_Reg_ cells which further suppress effector immune responses [15].

With this background, PD-1 signaling represents a viable target for novel anti-cancer therapy. The development and clinical application of immune checkpoint inhibitors significantly enhance antitumor immunity, produce durable responses, and prolong survival in cancer patients.

## Immune checkpoint inhibitors of PDL1 as cancer therapeutics

Immune checkpoint inhibitors, especially PD-1 and PD-L1 have shown clinical efficacies against many different solid and hematologic malignancies [28]. Binding of PD-L1 to its receptor suppresses T cell migration, proliferation, and secretion of cytotoxic mediators, and restricts tumor cell killing. Inhibitors of PD-1 and PD-L1 disrupt PD-1 axis thereby reverses T cell suppression and enhances endogenous antitumor immunity to unleash long-term antitumor responses for patients with a wide range of cancers [29]. In addition to binding PD-1, PD-L1 also interacts with B7 (CD80, CD86) creating negative signals on T cells and dampens antitumor immunity [28, 29].

Development and clinical use of PD-1 and PD-L1 inhibitors for anti-cancer therapy have broadened. Correspondingly, there is a range of commercially available immunohistochemistry (IHC)-based assays to detect the presence of the PD-L1 protein in tumors [30]. Those assays are divided into two types: companion diagnostics assays which provide information that is essential for safe and effective use of the corresponding drugs, and complementary (or co-diagnostic) assays which may be used in treatment selection, but are not considered essential for safe and effective use of the corresponding therapy. Of note, PD-L1 IHC22C3 pharmDx tests have status as a companion diagnostics and PD-L1 IHC 28-8 pharmDx, PD-L1 IHC 22C3 pharmDx, Ventana PD-L1 SP142, and Ventana PD-L1 SP2632 testing have status as complementary diagnostics [30–33].

Currently, there are three approved PD-L1 inhibitors by the US Food and Drug Administration (FDA) for cancer treatment ranging from non-small cell lung cancer to Merkel cell carcinoma.

Among many ongoing phase II/III trials of atezolizumab, durvalumab, and avelumab, this review also summarized new PD-L1 inhibitors in clinical developments.

## Atezolizumab

Atezolizumab, formerly known as MPDL3280, is a fully humanized IgG1 monoclonal antibody that is engineered with a modification in the Fc domain that eliminates antibody-dependent cellular cytotoxicity to prevent depletion of T cells expressing PD-L1. The compound blocks the interaction of PD-L1, specifically on tumor cells and tumor-infiltrating immune cells, with both PD-1 and B7.1, but not the interaction of PD-L2. In preclinical studies, atezolizumab have shown an increased level of proliferating CD8+ T cells by inducing cytokine changes including transient increases in IL-18, IFNγ, and CXCL11, as well as transient decrease in IL-6 [34, 35]. Through the inhibition of PD-L1, atezolizumab reduces immunosuppressive signals found within the tumor microenvironment and consequently increases T cell-mediated immunity against tumors [35].

Clinical data have demonstrated that atezolizumab is safe and efficacious in a wide range of solid tumors and hematologic malignancies. In a phase I dose-finding study, three dosing schedules of atezolizumab were tested in patients with recurrent non-small cell lung cancer (NSCLC), melanoma, renal cell carcinoma, colorectal cancer, gastric cancer, and head and neck squamous cell carcinoma [36]. Two hundred and seventy-seven patients were enrolled and received intravenous atezolizumab every 3 weeks (q3w) at doses of 10, 15, or 20 mg/kg of body weight until disease progression (PD) or unacceptable toxicity. Overall, atezolizumab was well tolerated up to the maximum administered dose of 20 mg/kg [36]. The most common adverse effects (AEs) were fatigue, decreased appetite, nausea, pyrexia, diarrhea, rash, pruritus, arthralgia, and headache. Of 175 patients evaluated for response, the overall response rate achieved (ORR) was 21% across all histological types. Similarly, Powles et al. in a phase I escalation and expansion study investigated the safety and activity of single-agent atezolizumab at a dose of 15 mg/kg of body weight every 3 weeks in 205 heavily pretreated patients with urothelial carcinoma (UC) which were stratified by the percentage of PD-L1-positive immune cells in the tumor microenvironment by immunohistochemistry (IHC) defined by IHC 0 (< 1%), IHC 1 (≥ 1% but < 5%), and IHC 2/3 (≥ 5%) [37]. After a median duration of 65 days of treatment, there were no dose-limiting toxicities (DLTs) occurred and atezolizumab was found to be safe and well tolerated in patients with UC. Most treatment-related AEs were grade 1 or 2 fatigue, nausea, decreased appetite, and pyrexia, and many were transient in nature. The ORRs were 43% for those with IHC 2/3 tumors and 11% for those with IHC 0 or 1 (0/1) tumors after a minimum of 6 weeks follow-up [37]. Based on these data, the FDA granted atezolizumab as a breakthrough status for UC.

Given the modest activity of atezolizumab in the precedent phase I studies, larger studies were needed to clarify its efficacy. Rosenberg and colleagues reported that atezolizumab at a fixed dose of 1200 mg administered on day 1 of each 21-day cycle demonstrated durable activity in heavily pretreated patients with inoperable locally advanced or metastatic urothelial carcinoma regardless of PD-L1 expression whose disease had progressed after previous platinum-based chemotherapy [38]. The final results of this phase II trial (IMvigor 210) showed ORR of 10% with best efficacy demonstrated in patients with increased levels of PD-L1 expression [38]. On the basis of these data, FDA granted accelerated approval to atezolizumab for locally advanced or metastatic urothelial carcinoma treatment after failure of cisplatin-based chemotherapy in May 2016. However, the confirmatory trial failed to achieve its primary endpoint of overall survival. In a phase III trial (IMvigor 211) by Powles et al. comparing atezolizumab vs chemotherapy (vinflunine 320 mg/m^2^, paclitaxel 175 mg/m^2^, or 75 mg/m^2^ docetaxel) every 3 weeks in patients with locally advanced or metastatic urothelial carcinoma after progression with platinum-based chemotherapy demonstrated that overall survival and ORRs were similar (median OS 11.1 months vs 10.6 months, hazard ratio [HR] 0.87; *p* = 0.41 and 23% vs 22%, respectively) [39]. Nevertheless, the duration of response was numerically longer in the atezolizumab group (15.9 months) than in the chemotherapy group (8.3 months) [39].

Atezolizumab was also FDA approved in October 2016 for metastatic NSCLC whose disease progressed during or following platinum-containing chemotherapy. The randomized phase II study (POPLAR) observed that atezolizumab met its primary endpoint and showed a statistically significant survival benefit compared to docetaxel (HR = 0.54; *p* = 0.014) in people with recurrent NSCLC whose tumors expressed medium and high levels of PD-L1 expression [40]. Likewise, a phase II study (BIRCH) met its primary endpoint and showed that atezolizumab shrank tumors with ORR of up to 22% in patients with previously untreated and patients whose disease had progressed on prior one or more chemotherapy including platinum-containing chemotherapy [41]. Encouraging results from the POPLAR and BIRCH studies prompted a phase III trial (OAK) evaluating the efficacy and safety of atezolizumab vs docetaxel in previously treated patients with non-small cell lung cancer [42]. Tolerability profile was acceptable, and adverse events were consistent with those observed in previous studies. The OAK trial met its co-primary endpoints and showed a statistically significant OS advantage with atezolizumab compared to docetaxel in intention-to-treat (ITT) and PD-L1 expression population (IHC 1/2/3) with median OS of 13.8 months vs 9.6 months and 15.7 months vs 10.3 months, respectively [42].

The promising results of single-agent atezolizumab have led investigators to explore the synergistic efficacy in combination with established chemotherapy regimen(s) [43–45].

The preliminary results from a phase III IMpower131 study showed that atezolizumab plus chemotherapy (carboplatin and *nab*-paclitaxel) reduced the risk of disease worsening or death (PFS) by 29% compared with chemotherapy (carboplatin and *nab*-paclitaxel) in previously untreated patients with advanced squamous NSCLC [46]. At the time of the interim analysis, the overall survival benefit was not observed and the study will continue as planned [46].

A phase III, IMpower132, open-label, randomized study evaluating the efficacy and safety of atezolizumab plus chemotherapy (cisplatin or carboplatin and pemetrexed) vs chemotherapy alone in chemotherapy-naive patients with advanced non-squamous NSCLC demonstrated reduction in the risk of disease worsening or death by 40% with atezolizumab plus chemotherapy compared with chemotherapy alone (PFS = 7.6 vs 5.2 months; hazard ratio [HR] = 0.60; *p* < 0.0001) [47]. Although a numerical improvement for the co-primary endpoint of overall survival was observed, statistical significance was not met at this interim analysis and the study will continue as planned [47].

The phase III IMpower130 study met its co-primary endpoint of progression-free survival and overall survival with atezolizumab plus chemotherapy (carboplatin plus nab-paclitaxel) compared to chemotherapy alone as the first-line treatment of patients with stage IV non-squamous non-small cell lung cancer and no *ALK* or *EGFR* mutations [48]. There were significant improvements in the median overall survival (18.6 months vs 13.9 months) and the median progression-free survival (7.0 months vs 5.5 months) in the atezolizumab plus chemotherapy group and chemotherapy group alone, respectively [48].

In December 2018, atezolizumab in combination with bevacizumab and standard chemotherapy for the first-line treatment of patients with metastatic non-squamous, non-small cell lung cancer (NSq NSCLC) with no *EGFR* or *ALK* genomic tumor aberrations was approved by the FDA. This was based on the IMpower150 trial, an open-label, randomized (1:1:1), three-arm study enrolling 1202 patients receiving atezolizumab, carboplatin, paclitaxel, and bevacizumab (4-drugregimen) vs atezolizumab, carboplatin and paclitaxel (3-drug regimen) vs carboplatin, paclitaxel, and bevacizumab (control arm) until disease progression or unacceptable toxicity [43]. The addition of atezolizumab to bevacizumab and standard chemotherapy demonstrated OS advantage with median OS of 19.2 months and 14.7 months for those receiving carboplatin, paclitaxel, and bevacizumab regardless of PD-L1 expression and *EGFR* or *ALK* genetic alteration status. Progression-free survival was also longer in the 4-drug regimen than in the control arm [43].

In a similar fashion, atezolizumab in combination with standard chemotherapy for the first-line treatment of patients with extensive-stage small cell lung cancer was granted approval by the FDA in March 2019. Four hundred three treatment-naïve patients with extensive-stage small cell lung cancer were randomly assigned in a 1:1 ratio to receive carboplatin and etoposide with either atezolizumab or placebo for four 21-day cycles (induction phase), followed by a maintenance phase during which they received either atezolizumab or placebo until unacceptable toxic effects, disease progression, or no additional clinical benefit [44]. This IMpower133 trial met its co-primary endpoints. At a median follow-up of 13.9 months, the PFS and OS of patients who received carboplatin and etoposide with atezolizumab were significantly longer (5.2 months; 12.3 months) than those who received chemotherapy regimen alone (4.3 months; 10.3 months) [44]. The most common adverse events reported in ≥ 20% of patients who received atezolizumab were fatigue/asthenia, nausea, alopecia, constipation, and decreased appetite.

Likewise, Schmid et al. evaluated the efficacy and safety of nab-paclitaxel with or without atezolizumab in 451 patients with treatment-naïve metastatic triple-negative breast cancer (TNBC) until disease progression or unacceptable toxicities [45]. At a median follow-up of 12.9 months, the addition of atezolizumab to nab-paclitaxel led to a 40% reduction in the risk of progression or death compared with nab-paclitaxel alone among patients with PD-L1-positive tumors [45]. Safety analysis demonstrated no new adverse effects. On the basis of these data, FDA granted accelerated approval to the frontline combination of atezolizumab plus nab-paclitaxel for patients with unresectable locally advanced or metastatic PD-L1-positive TNBC in March 2019.

Of note, immune checkpoint inhibitors including atezolizumab are only effective in a subset of patients, and some patients who respond initially show a subsequent rapid disease progression due to primary and acquired resistance to PD-1/PD-L1 inhibition. New strategies are being explored to prevent or reverse resistance to therapy leading to improved patient outcomes. Recent studies suggest that targeted therapy enhance antitumor immune responses by releasing new antigens and provide a basis for immunotherapy combined with targeted therapy [49–51]. In a phase Ib study of atezolizumab in combination with erlotinib in treatment-naïve epidermal growth factor receptor (EGFR) mutation-positive advanced NSCLC, the ORR was 75% and median PFS was 15 months. The median OS was not reached [52]. Additional phase I–III clinical trials are underway to test atezolizumab with or without chemotherapy in the setting of advanced or metastatic solid tumors and hematological malignancies including melanoma, prostate cancer, pancreatic cancer, renal cell carcinoma, colorectal cancer, gastric cancer, ovarian cancer, and multiple myeloma [53].

## Durvalumab

Durvalumab, also known as MEDI4736, is a fully human IgG1 monoclonal antibody that binds with high affinity and specificity to PD-L1, blocking the interaction with PD-1 and CD80 molecules [54, 55]. The compound is uniquely engineered to prevent antibody-dependent cell-mediated cytotoxicity on T cells expressing PD-L1. Durvalumab is a potent inhibitor with subnanomolar activity [PD-1 (IC_50_ = 0.1 nM) and CD80 (IC_50_ = 0.04)] against PD-L1 [54]. In vivo studies revealed that durvalumab significantly inhibits the growth of human tumors in a novel xenograft model containing co-implanted human T cells [55]. Given these promising preclinical data, durvalumab was advanced into clinical development. The pharmacokinetics (PK), safety, and tolerability of durvalumab were first evaluated in a phase I study of 32 patients with advanced solid tumors by employing a dose-escalating design [56]. Overall, a dose of 10 mg/kg of body weight every 2 weeks of durvalumab was selected for future studies [56]. Updated results from an ongoing open-label, dose-escalation, dose-expansion trial (study 1108) showed that durvalumab demonstrates favorable clinical activity in patients with advanced urothelial bladder carcinoma [57–59]. Durvalumab was well tolerated at a dose of 10 mg/kg every 2 weeks for up to 12 months or until progression, starting another anticancer therapy, or unacceptable toxic effects [58, 59]. Tolerability of durvalumab was similar to other PD-L1 inhibitors with most common toxicities including fatigue, diarrhea, and decreased appetite [58]. Responses were early, durable, and observed regardless of PD-L1 expression with higher ORR of 27.6% in patients with high and low expression of PD-L1 compared to 5.1% in patients with negative expression of PD-L1 [59]. On May 1, 2017, the FDA granted accelerated approval to durvalumab for the treatment of patients with locally advanced or metastatic urothelial carcinoma who have disease progression during or following platinum-containing chemotherapy or who have disease progression within 12 months of neoadjuvant or adjuvant treatment with platinum-containing chemotherapy. This accelerated approval of durvalumab in bladder cancer is contingent upon results from an ongoing, phase III DANUBE confirmatory trial as a frontline therapy and combination with tremelimumab for patients with metastatic urothelial carcinoma, regardless of their eligibility for cisplatin-based chemotherapy.

Durvalumab was also FDA approved on February 16, 2018, for patients with unresectable stage III NSCLC whose disease has not progressed following concurrent platinum-based chemotherapy and radiation therapy based on the results from the PACIFIC trial [60]. It is the first study that demonstrates a survival advantage for unresectable, stage III non-small cell lung cancer. In this randomized, double-blinded, placebo-controlled, multicenter phase III study, durvalumab as a consolidation therapy was compared with placebo in patients who received two or more cycles of platinum-based chemotherapy concurrently with definitive radiation therapy. The trial met its co-primary endpoints with 2-year OS rates in the durvalumab group, 66.3% compared with 55.6% for patients who were treated with placebo. The median PFS was 17.2 months and 5.6 months in the durvalumab group and placebo group, respectively [60]. The median overall survival was not reached in the durvalumab arm and was 28.7 months in the placebo arm. The updated analysis published in the Journal of Clinical Oncology in May 2019 demonstrated that the 36-month OS rates with durvalumab and placebo were 83.1% and 74.6%, respectively [61].

Given the single-agent activity of durvalumab in previous studies, testing of this compound in combination with chemotherapy, immunotherapy, and targeted therapy was seen as a logical step to maximize benefit. Rizvi and colleagues evaluated 1118 previously untreated patients with metastatic NSCLC who were randomly assigned to durvalumab alone, durvalumab plus tremelimumab (anti-cytotoxic T lymphocyte-associated protein 4 [CTLA4] antibody), or chemotherapy [62]. This phase III MYSTIC study failed to meet its co-primary endpoint of progression-free survival and overall survival. The final data showed that OS was 11.9 vs 12.9 months (HR 0.85; *p* = 0.202) for patients with PD-L1 expression ≥ 25% who received durvalumab/tremelimumab vs standard platinum-based chemotherapy, respectively [62]. The median PFS was 3.9 months vs 5.4 months (HR 1.05; *p* = 0.705) for patients who received durvalumab/tremelimumab vs standard platinum-based chemotherapy, respectively [62].

Correspondingly, the addition of tremelimumab to durvalumab did not meet the primary endpoint of improving OS compared to standard chemotherapy in patients with recurrent or metastatic head and neck squamous cell carcinoma (HNSCC) who progressed after platinum-based chemotherapy whose tumors express PD-L1 on 25% or more of their cancer cells [63]. This open-label phase III EAGLE trial randomized patients to single-agent durvalumab, durvalumab plus tremelimumab, or standard-of-care chemotherapy [63]. While the EAGLE trial did not meet the primary endpoint, the results of the phase III KESTREL trial of durvalumab and tremelimumab in patients who have not received prior chemotherapy for recurrent or metastatic HNSCC are awaited in the first half of 2019 [63].

There is also a rationale for combining immunotherapy plus targeted therapy to improve patients’ outcome [49–51]. A phase Ib TATTON trial of durvalumab plus osimertinib for first- and second-line treatment of EGFR-positive NSCLC demonstrated a high incidence of interstitial lung disease [64], and the study was suspended.

Currently, there are many studies in different phases of durvalumab alone or in combination with chemotherapy and/or immunotherapy for patients with advanced solid tumor and hematologic malignancies including NSCLC, ovarian cancer, esophageal cancer, renal cell carcinoma, mantle cell lymphoma, diffuse large B cell lymphoma, and follicular lymphoma.

## Avelumab

Avelumab (MSB0010718C) is another fully human IgG1 monoclonal antibody that specifically binds to PD-L1, preventing the interaction between PD-L1 and the inhibitory T cell receptors, PD-1 and B7.1 resulting in T cell-mediated, adaptive antitumor immune responses and T cell reactivation and cytokine production [28, 65]. Unlike atezolizumab and durvalumab, avelumab has a wild-type IgG1 crystallizable fragment (Fc) region, which enables the compound to engage with Fc-γ receptors on natural killer cells and induce tumor-directed antibody-dependent cell-mediated cytotoxicity (ADCC) in preclinical studies [66, 67]. Therefore, avelumab utilizes both adaptive and innate immune mechanisms to destroy cancer cells.

The dose-escalation phase I study (JAVELIN Solid Tumor) by Heery et al. indicated that avelumab was generally well tolerated at doses ranging from 1 to 20 mg/kg body weight every 2 weeks in patients (*n* = 53) with metastatic or locally advanced previously treated solid tumors [68]. The common treatment-related adverse events include fatigue, influenza-like symptoms, fever, and chills. There was only one DLT occurred at a dose of 20 mg/kg; therefore, a dose of 10 mg/kg every 2 weeks was selected for future studies [68]. Preliminary results from JAVELIN Merkel 200 trial of avelumab had been recently reported [69]. In this first phase II study, 88 patients with stage IV chemotherapy-refractory, Merkel cell carcinoma (MCC) treated with avelumab at a dose of 10 mg/kg body weight every 2 weeks were enrolled. At a median of 10.4 months, avelumab was well tolerated and active, with an ORR of 33% with 11% complete and 22% partial response rates, and median PFS was 2.7 months [69]. The duration of response ranged from 2.8 to 23.3+ months with 86% of responses durable for 6 months or longer regardless of the PD-L1 expression status. On the basis of these data, FDA granted accelerated approval to avelumab for the treatment of patients 12 years and older with metastatic MCC in March 2017.

Likewise, avelumab was granted accelerated approval for the treatment of patients with locally advanced or metastatic urothelial carcinoma with disease progression during or following platinum-containing chemotherapy, or within 12 months of neoadjuvant or adjuvant platinum-containing chemotherapy in May 2017. This was based on the data from the urothelial carcinoma cohorts of the phase Ib JAVELIN Solid Tumor trial [70, 71], in which the ORR was 13.3% among 226 patients who had been followed for at least 13 weeks and was 16.1% among 161 patients who had been followed for at least 6 months. The median response duration had not been reached.

A phase III JAVELIN Ovarian 100 study exploring frontline avelumab in ovarian cancer has been terminated because of independent panel determination that the study would not meet its primary endpoint of PFS [72]. Likewise, the phase III JAVELIN Ovarian 200 trial [73] showed that avelumab alone or in combination with pegylated liposomal doxorubicin (PLD) did not induce a statistically significant improvement in OS or PFS vs PLD alone in patients with platinum-resistant/refractory ovarian cancer and failed to meet the primary endpoints of the study [73]. Avelumab was also studied in advanced gastric cancer/gastro-esophageal junction cancer (GC/CEJC) in whom two prior lines of therapy have failed. A phase III JAVELIN Gastric 300 trial compared avelumab to physician’s choice of chemotherapy as the third-line therapy [74]. The primary end of improving OS was not met along with secondary endpoints of PFS or ORR [74]. Barlesi et al. investigated the efficacy and safety of avelumab in patients with NSCLC who had already received platinum-based therapy [75]. Compared with docetaxel, avelumab did not improve the overall survival in patients with platinum-treated PD-L1-positive NSCLC but had a favorable safety profile [75]. Updated interim results from an ongoing, open-label phase Ib trial showed that avelumab is active in patients with metastatic triple-negative breast cancer (TNBC) with higher response rate observed in PD-L1-positive tumors [76]. In a recent report of ongoing phase Ib cohort study of avelumab demonstrated durable responses, promising survival outcomes, and an acceptable safety profile in heavily pretreated patients with metastatic melanoma [77].

The combination of avelumab and axitinib, a VEGF receptor inhibitor, has been evaluated in a phase IB treatment-naive patients with advanced renal cell carcinoma [78]. The doublet therapy demonstrated acceptable safety profile and early sign of clinical activity. The maximum tolerated dose established for the combination was avelumab 10 mg/kg every 2 weeks and axitinib 5 mg twice daily [78]. In light of these encouraging results, a phase III JAVELIN Renal 101 trial conducted by Motzer and colleagues randomized 886 previously untreated patients with advanced or metastatic renal cell carcinoma in a 1:1 ratio to receive avelumab at 10 mg/kg every 2 weeks plus axitinib at 5 mg orally twice daily or sunitinib 50 mg orally once daily for 4 weeks (6-week cycle) [79]. Median PFS was significantly longer with avelumab plus axitinib (13.8 months) than sunitinib (8.4 months) in the overall population [79]. Moreover, the ORR with avelumab/axitinib was 51.4% and 25.7% with sunitinib. Based on those findings, the FDA approved the combination of avelumab and axitinib for the frontline treatment of patient with advanced renal cell carcinoma in May 2019.

Other phase I–III trials are currently ongoing in a variety of tumor types.

## Envafolimab

Envafolimab (also known as KN 035 and ASC 22) is a first-in-class nanobody (single domain antibody) created by a fusion of the of anti-PD-L1 domain with Fc fragment of human IgG1 antibody that binds with high affinity and specificity to PD-L1, blocking interaction with PD-1, and resulting in T cell-mediated immune response to neoplasms. In biochemical assays, envafolimab blocks interaction between PD-L1 and PD-1 with an IC_50_ value of 5.25 nm in a competitive ELISA [80]. In contrast to other PD-L1 inhibitors, envafolimab is administered as a subcutaneous injection and demonstrates low immunogenicity and better penetration in tumor tissue in animal studies. In in vitro studies, the compound demonstrates dose- and time-dependent induction of T cell cytokine production in a mixed lymphocyte reaction [80]. In xenograft models, envafolimab shows potent antitumor activity at comparable dosages (0.1–0.5 mg kg^−1^) [80]. In a phase I dose-escalation study, Papadopoulos and colleagues reported that envafolimab exhibited favorable safety profile and preliminary evidence of encouraging anti-tumor activity in patients with advanced solid tumors [81]. The compound was given subcutaneously at a dosage of 0.01, 0.03, 0.1, 0.3, 1.0, 2.5, 5.0, and 10.0 mg/kg weekly. There was no DLT observed in this trial. Based on these results, a phase II study for microsatellite instability-high (MSI-H) advanced solid tumors and a phase III trial for patients with biliary tract carcinoma (cholangiocarcinoma) have been initiated in China.

## BMS-936559

BMS-936559 (also known as MDX-1105) is a high-affinity fully humanized IgG4 monoclonal antibody that specifically inhibits PD-L1 binding to both PD-1 and CD80.

The safety and clinical activity of BMS-936559 were undertaken in a phase I study of 207 patients with advanced solid tumors by employing a dose-escalating design (0.3–10 mg/kg every 14 days, 3 times in each 6-week course) for up to 16 cycles [82]. The compound was well tolerated with most common drug-related adverse events including fatigue, infusion reactions, diarrhea, arthralgia, rash, nausea, pruritus, and headache. Early results at a median duration of 12 weeks showed that ORR was 6–17% with prolonged stabilization of disease in patients with advanced cancers, including non-small cell lung cancer, melanoma, and renal cell cancer [82].

## CK-301

CK-301 is a fully human monoclonal antibody of IgG1 subtype that directly binds to PD-L1 and blocks its interactions with PD-1 and B7.1 receptors. Similar to avelumab, CK-301 has functional Fc domain and is capable of inducing ADCC and complement-dependent cytotoxicity (CDC)-mediated killing of PD-L1+ cell lines, including lymphoma cells [83]. In the cellular assay, the compound exhibited subnanomolar binding affinity for PD-L1 with increased production interferon-gamma by primary human T cells in mixed lymphocyte reaction (MLR) culture [83]. A first-in-human phase 1, open-label, multicenter, dose-escalation study of CK-301 administered intravenously as a single agent for patient with advanced cancers is ongoing (NCT03212404).

## CS-1001

As a novel, highly potent, first-in-class full-length IgG4 monoclonal antibody, CS-1001 selectively binds to PD-L1 and blocks interaction with PD-1, resulting in T cell-mediated antitumor immune responses. A phase I study examined the safety, tolerability, PK, and anti-tumor activity of CS-1001 in patients with advanced tumors. The compound was dosed every 3 weeks across five dose-escalating cohorts of 3 mg/kg, 10 mg/kg, 20 mg/kg, 40 mg/kg, and 1200 mg [84]. CS-1001 was generally well tolerated with linear PK profile, and most frequent treatment-emergent AEs were grade 1/2 anemia, nausea, decreased appetite, leucopenia, and proteinuria. It also exhibited antitumor activity with a disease control rate (DCR) of 58% [84].

A phase III GEMSTONE-303 study evaluating the efficacy and safety of CS1001 plus oxaliplatin and capecitabine (XELOX) chemotherapy for the first-line treatment in patients with unresectable, locally advanced, or metastatic gastric adenocarcinoma or GEJ adenocarcinoma is currently accruing. Additionally, there are two pivotal phase II studies for patients with relapsed/refractory extranodal natural killer/T cell lymphoma (NKTL) (NCT03595657) and relapsed/refractory classical Hodgkin lymphoma (rr-cHL)(NCT03505996) and two phase III studies for patients with stage IV non-small cell lung cancer (NCT03789604) and locally advanced/unresectable (stage III) non-small cell lung cancer that has not progressed after prior concurrent/sequential chemoradiotherapy (NCT03728556) have been initiated in China.

## SHR-1316 (HTI-1088)

SHR-1316, a fully humanized IgG4 monoclonal antibody, binds specifically to human PD-L1 and blocks the interaction of PD-L1 on cancer cells with its receptor PD-1 on T cells and mediating antitumor immune responses. An open-label, non-randomized, dose escalation/expansion phase I study of SHR-1316 for patients with advanced/metastatic solid tumors who have failed current standard anti-tumor therapies has been initiated and accrued participants in china (NCT03133247).

## CBT-502 (TQB-2450)

CBT-502 is another novel, fully humanized IgG1 monoclonal antibody against PD-L1 developed by CBT Pharmaceuticals, Inc. In the cellular assay, the compound effectively blocked the interaction of PD-L1 with PD-1 and PD-L1 with CD80 at a concentration (IC_50_) of 47.97 pM and 1.09 nM, respectively, and strongly activated T cells by the production of IFN-gamma in a mixed lymphocyte reaction [85]. In in vivo studies, CBT-502 showed potent antitumor activity in a dose-dependent manner in the MC-38/H-11 murine colon and A375 human melanoma animal models [85]. With these promising preclinical data, CBT-502 is undergoing clinical development to assess its safety and tolerability in patients with advanced tumors (NCT03825705, NCT03800706, NCT03855384).

## BGB-A333

BGB-A333 is a fully humanized IgG1-variant monoclonal antibody that specifically target and binds to PD-L1, blocking interaction to its receptor, PD-1 on T cell, reversing T cell inactivation, and increases T cell expansion resulting in cytotoxic T cell-mediated antitumor immune response against PD-L1-expressing tumor cells [86]. The compound also inhibits PD-L1-induced apoptosis of activated CD8+ T cells and increases T cell proliferation [86]. An early phase I/II study investigating the safety, tolerability, pharmacokinetics, and preliminary antitumor activity of BGB-A333 alone and in combination with anti-PD-1 monoclonal antibody, tislelizumab, in patients with advanced solid tumors is accruing participants [86].

## Conclusions and future directions

Despite early/accelerated FDA approval, many confirmatory phase III trials have failed to meet the primary endpoints including PFS or OS. The field of immunotherapeutic is relatively new and has yet to reach its prime. Furthermore, serious investigation into predictive biomarkers, mechanism of resistance, PD-L1 expression threshold, more sensitive biomarker assays, immune-related toxicity, and treatment duration are needed. Though cancer patients can be resistant to immune checkpoint inhibitors, other immunotherapy targeting cytokines, tumor-directed antibodies, antibody-drug conjugates, chimeric antigen receptor (CAR) T cells therapy, vaccines, and even genetic therapy are paving way for more tumor-specific therapy. Currently, there are many PD-L1 inhibitors that are undergoing various clinical trials, and three have been granted FDA approval. There is a comprehensive list of PD-L1 inhibitors in the current phases of clinical trials in Table 1. Current FDA-approved PD-L1 inhibitors with indications and dosages are listed in Tables 1 and 2.

**Table 1:** Comprehensive list of PD-L1 inhibitors along with FDA-approved disease treatment, clinical trials, and primary endpoints

PD-L1 inhibitors/company	Disease	Drug therapy	Primary endpoints	References
Atezolizumab (MPDL3280A), Roche Genentech	NSCLC	Atezolizumab vs docetaxel	OS, 15.7 months vs 10.3 months	[42]
	NSq NSCLC	Atezolizumab, carboplatin, paclitaxel, and bevacizumab vs atezolizumab; carboplatin and paclitaxel vs carboplatin, paclitaxel, and bevacizumab	OS 19.2 months (4 drugs) vs 14.7 months (3 drugs)	[43]
	UC	Atezolizumab vs chemotherapy	OS 11.1 months vs 10.6 months	[37, 38]
	ES-SCLC	Atezolizumab, carboplatin, and etoposide vs carboplatin and etoposide	PFS 5.2 months vs 4.3 months; OS 12.3 months vs 10.3 months	[44]
	TNBC	Atezolizumab plus nab-paclitaxel vs nab-paclitaxel	PFS 7.5 months vs 5.0 months; OS 25.0 months vs 15.5 months	[45]
Durvalumab (MEDI4736), AstraZeneca	UC	Durvalumab	ORR 17.8%	[59]
	Unresectable stage III NSCLC	Durvalumab vs placebo	PFS 16.8 months vs 5.6 months	[60]
Avelumab (MSB0010718C), Merck and Pfizer	MCC	Avelumab	ORR 33% (11% complete and 22% partial)	[69]
	UC	Avelumab	ORR 16.1% at least 6 months	[70, 71]
	RCC	Avelumab plus axitinib vs sunitinib	PFS 13.8 months vs 8.4 months	[79]
Envafolimab (KN035), Alphamab Oncology	N/A	N/A		[80, 81]
BMS-936559, Bristol-Myers Squibb	N/A	N/A		[82]
CK-301**,**Checkpoint Therapeutics	N/A	N/A		[83]
CS-1001, CStone Pharmaceuticals	N/A	N/A		[84]
SHR-1316 (HTI-1088), Hengrui Therapeutics	N/A	N/A		[85]
BGB-A333, BeiGene	N/A	N/A		[86]

*NSCLC* non-small cell lung cancer, *UC* urothelial cancer, *MCC* Merkel cell carcinoma, *TNBC* triple-negative breast cancer, *RCC* renal cell carcinoma, *ES-SCLC* extensive-stage small cell carcinoma, *NSq NSCLC* non-squamous non-small cell lung cancer, *MUC* metastatic urothelial carcinoma, *PFS* progression free survival, *OS* overall survival, *ORR* objective response rate

**Table 2:** Current list of FDA-approved PD-L1 inhibitors along with indications, dosages, and bioassay for the PD-L1 expression

PD-L1 inhibitors	Indications	Dosages and schedules	PD-L1 bioassays	PD-L1 cutoff	References
Atezolizumab (MPDL3280A)	NSCLC	1200 mg over 60 min q3 weeks	Roche Ventana SP142	≥ 50% TC or ≥ 10% IC	[42]
	UC			≥ 5% IC	[38]
	ES-SCLC			N/A	[44]
	TNBC	840 mg q1 and 15		≥ 1% IC	[45]
Durvalumab (MEDI4736)	UC	10 mg/kg over 60 min q2 weeks	Roche Ventana SP263	≥ 25% TC or ≥ 25% IC	[59]
	Unresectable stage III NSCLC			≥ 1% TC	[60]
Avelumab (MSB0010718C)	MCC	10 mg/kg over 60 min q2 weeks	N/A	N/A	[69]
	RCC				[79]

*NSCLC* non-small cell lung cancer, *UC* urothelial cancer, *MCC* Merkel cell carcinoma, *TNBC* triple-negative breast cancer, *RCC* renal cell carcinoma, *ESSCLC* extensive-stage small cell carcinoma, *% IC* percentage PD-L1 expressing tumor-infiltrating immune cells of any intensity of the tumor area, *% TC* percentage of PD-L1 expressing tumor cells of any intensity

**Table 3:** Comprehensive list of PD-L1 inhibitors and PD-1 inhibitors with antibody class and immune adverse events. This table shows the similarities and differences between PD-1/PD-L1 inhibitors

Target	Drugs	Antibody class	Immune-related adverse events
PD-L1	Atezolizumab	IgG1	Pneumonitis, hepatitis, colitis, endocrinopathies (thyroid disease, adrenal insufficiency, hypophysitis, type 1 diabetes), meningitis/encephalitis, pancreatitis, dermatitis/rash
	Durvalumab	IgG1	Pneumonitis, hepatitis, colitis, endocrinopathies (thyroid disease, adrenal insufficiency, hypophysitis, type 1 diabetes), nephritis
	Avelumab	IgG1	Pneumonitis, hepatitis, colitis, endocrinopathies, nephritis, renal dysfunction
	Envafolimab	IgG1	Increased aspartate aminotransferase, increased alanine aminotransferase, lymphopenia
	BMS-936559	IgG4	Hypothyroidism, hepatitis, sarcoidosis, endophthalmitis, diabetes mellitus, myasthenia gravis
	CK-301	IgG1	N/A (ongoing)
	CS-1001	IgG4	Anemia, increased blood bilirubin, protein urine present, white blood cell count decreased, proteinuria
	SHR-1316	IgG4	N/A (ongoing)
	CBT-502	IgG1	N/A (ongoing)
	BGB-A333	IgG1 variant	N/A (ongoing)
PD-1	Nivolumab	IgG4	Pneumonitis, hepatitis, colitis, endocrinopathies, nephritis, renal dysfunction, encephalitis, rash
	Pembrolizumab	IgG4	Pneumonitis, hepatitis, colitis, endocrinopathies, nephritis, renal dysfunction
	Cemiplimab	IgG4	Cellulitis, pneumonitis, hypercalcemia, pleural effusion

A comprehensive list of PD-L1 & PD-1 inhibitors along with antibody class and immune adverse events are listed in Table 3.
